# Effects of black soldier fly larvae (*Hermetia illucens* L.) as feed supplements on muscle nutrient composition, meat quality, and antioxidant capacity in Qianbei goat

**DOI:** 10.5713/ab.24.0173

**Published:** 2024-06-25

**Authors:** Shengyong Lu, Siwaporn Paengkoum, Shengchang Chen, Yong Long, Xinran Niu, Sorasak Thongpea, Nittaya Taethaisong, Weerada Meethip, Pramote Paengkoum

**Affiliations:** 1 School of Animal Technology and Innovation, Institute of Agricultural Technology, Suranaree University of Technology, Muang, Nakhon Ratchasima 30000, Thailand; 2 Program in Agriculture, Faculty of Science and Technology, Nakhon Ratchasima Rajabhat University, Muang, Nakhon Ratchasima, 30000, Thailand; 3 Institute of Animal Nutrition and Feed Science, Guizhou University, Guiyang 550025, China

**Keywords:** Antioxidant Capacity, Black Soldier Fly Larvae, Goat, Heat Treatment Black Soldier Fly, Meat Quality

## Abstract

**Objective:**

Black soldier fly (BSF) as an animal protein feed source is currently becoming a hot research topic. This study investigated the effects of the BSF as a protein feed source for goats on slaughter performance, muscle nutrient composition, amino acids, fatty acids, minerals, and antioxidant levels.

**Methods:**

Thirty Qianbei Ma goats (20.30±1.09 kg) were randomly divided into three groups: the control group (GRP_C_) supplemented with 10% full-fat soybean, treatment 1 (GRP_U_) supplemented with 10% untreated BSF, and treatment 2 (GRP_T_) supplemented with 10% heat-treated BSF. One-way analysis of variance among groups (with Fisher’s least significant difference post hoc comparison) was used in this study.

**Results:**

The nutrients, amino acids, fatty acids, minerals, and antioxidants in muscle were analyzed. The results showed that there were no significant differences in the moisture, dry matter, crude protein, ash, amino acids, and mineral content of the muscles among the three feeding groups. The slaughter rate and carcass weight of the GRP_U_ and GRP_T_ groups were significantly lower (p<0.05). The overall meat quality of the GRP_U_ and GRP_T_ groups decreased (p<0.05). The individual unsaturated fatty acids and total unsaturated fatty acids in the GRP_U_ group were higher (p<0.05) than those in the GRP_C_ and GRP_T_ groups. Both GRP_U_ and GRP_T_ decreased (p<0.05) the antioxidant capacity of the meat.

**Conclusion:**

Therefore, the heat-treated BSF had a better effect on meat quality compared to untreated BSF, but there were greater negative effects on the meat quality of GRP_U_ and GRP_T_ than GRP_C_.

## INTRODUCTION

Due to the projected 76% increase in global demand for meat, it is imperative to explore new methods for augmenting protein production while minimizing the utilization of natural resources to ensure food security within the carrying capacity of Earth [1]. Hence, there is a particular need to identify additional protein-rich feed ingredients for livestock with less environmental impact [2]. In this context, insects have been explicitly recommended as sustainable protein sources for both human consumption and livestock feed [3]. Currently, insects such as black soldier fly larvae (*Hermetia illucens*, L. BSF), crickets (Orthoptera: *Gryllidae*), and mealworm larvae (*Tenebrio molitor* L.) are being utilized as protein sources in animal feed [4]. These three insects are rich in protein and essential amino acids (EAA), minerals, fats, and a significant amount of saturated fatty acids (FA). Among other insect species, BSF stands out due to its excellent protein conversion rate [5]. However, a high content of saturated fatty acids (SFA) may have adverse effects on the rumen fermentation, and meat quality of goats [6]. Although the FA composition of ruminant tissues is less influenced by dietary lipid components compared to non-ruminant animals, differences in dietary FA composition can still lead to variations in tissue FA composition in goats [7]. This difference primarily arises from the hydrogenation of dietary lipids by rumen microorganisms, n-3 and n-6 FA from feed can be incorporated into the adipose tissues and muscles of ruminant animals [8]. Moreover, the levels of n-3 polyunsaturated fatty acids (PUFA) in ruminant tissues can be increased by feeding a diet rich in dietary lipids, which can be protected from rumen biohydrogenation through chemical or physical processing [9]. Previous research on rapeseed indicated that heat treatment reduced the biohydrogenation of unsaturated fatty acids (UFA) in the rumen, which subsequently increased the proportion of undegraded protein in the rumen [10]. In a study by Scollan et al [8], feeding a protected lipid supplement composed of soybean, flaxseed, and sunflower seeds resulted in a slight increase in the concentration of 18:3 n-3 in muscle phospholipids, rising from 12.7 mg/100 g to 16.0 mg/100 g, but there was no occurrence of chain elongation or desaturation of long-chain n-3 PUFA. Simultaneously, feeding heat-treated protein supplements can also enhance the tenderness of meat and intramuscular fat [11]. We hypothesized that heat-treated BSF could yield similar results. This experiment represents the final stage of a series of three trials. Earlier, we supplemented goat diets with varying levels of BSF and investigated the effects of different temperatures on BSF rumen degradation and protein digestion in the small intestine. Based on the above research, we determined the appropriate supplementation level and treatment temperature of BSF, and therefore, we conducted this experiment.

Guizhou Province is in the eastern part of the Yunnan-Guizhou Plateau in southwestern China, where there is lack of protein feed sources. The cultivation of BSF does not require extensive land, significant labor costs, or time investments, making it particularly suitable for development in countries and regions with limited arable land. The Qianbei Ma Goat (the goats raised in the north of Guizhou Province) is known for its excellent meat quality, with mild odor and tender texture [12]. Currently, it is not clear whether BSF has any negative impact on the meat quality of Qianbei Ma goat. Therefore, this study investigated the effects of heat-treated BSF on the slaughter performance, muscle nutritional composition, amino acid (AA), FA, minerals, and antioxidant levels of Qianbei Ma goat, providing data support for the application of BSF in goats.

## MATERIALS AND METHODS

### Ethics approval and consent to participate

Ethical permission for this study was obtained from the Experimental Animal Ethics Committee of Guizhou University (protocol number EAE-GZU-2021-E024).

### Animals, diets, and experimental design

BSF purchased from farmers in Henan Province, China, was ground and sieved through a 1 mm sieve to prepare the experimental powder. The chemical composition and minerals of BSF are listed in Table 1. This study was conducted at Fuxing Husbandry Co., Ltd., Guizhou, China (106.198244 E, 28.26403 N). After acclimatizing animals for 14 days, we tested them for the following 60 days. Thirty Qianbei Ma goats (20.30±1.09 kg) were randomly divided into three groups, and all of goats were fed individually. The control group (GRP_C_) was supplemented with 10% full-fat soybean, treatment 1 (GRP_U_) was supplemented with 10% untreated BSF, and treatment 2 (GRP_T_) was supplemented with 10% heat-treated BSF. Before formulating the feed, cassava was mixed with either full-fat soybean (FFS) or BSF in a 95:5 ratio. For the GRP_T_, an additional 75% water was added. The mixture was vigorously kneaded and firmly pressed evenly into a heated square pan (100 cm×55 cm×6.5 cm) to a thickness of about 5 cm, and treated in an oven at 140°C for 120 min. The experimental diets for goats were formulated according to NRC2007, as detailed in Table 2. Equal amounts of feed were offered daily at 9 a.m. and 5 p.m. for *ad libitum* water and intake, and 10% refusals on an as-fed basis. AA and FA in the diet were detailed respectively in Table 3 and 4.

### Chemical composition of feed and muscle

The BSF and basal diet were collected, dried in a vacuum oven at 65°C for 72 h, and further analyzed through a 1 mm sieve. Additionally, the *longissimus thoracis* and *lumborum* (LTL) (a muscle) were collected and lyophilized. The BSF, diet, and muscle were then analyzed for dry matter (DM), crude protein (CP), ether extract (EE), neutral detergent fiber (NDF), acid detergent fiber (ADF), and ash content, following AOAC method [13] methods. Mineral content in the feed, BSF, and LTL muscle was also analyzed using (AOAC 2005) [13] methods. Gross energy was determined using an adiabatic cartridge calorimeter (WGR-WR3; Changsha Benyi Instrument Co., Ltd., Changsha, China), with each sample assayed three times.

### Slaughtering procedure, carcass dissection and meat sampling

Slaughter and dissection procedures were conducted according to the Livestock and Poultry Slaughter Operation Regulations (Goat) (NY/T 3469-2019). Six goats were selected randomly to be weighed and slaughtered in each group after a 12-hour fasting period. The LTL muscle was separated from each carcass for meat quality testing. The dressing percentage (%) was calculated as (carcass weight/live weight before slaughter) ×100. The pH of the LTL muscle was measured at 45 min and 24 h post-mortem (maintained at 4°C) using a pH meter (Matthäus, Eckelsheim, Germany) that had been calibrated with standard pH 4.0 and 7.0 buffers. Meat color attributes, including lightness (L*), redness (a*), and yellowness (b*), were assessed using a portable meat colorimeter (NR110; Qinyou Science and Technology Development Co., Ltd., Chongqing, China). The colorimeter was pre-calibrated with a manual white calibration plate provided by the manufacturer, with an illuminance level of D65, an 8-degree standard viewer, and an aperture size of 4.0 mm for the closed candle. To determine water loss, fresh meat samples were cut and weighed after 45 minutes from slaughter (W3), covered with 17 layers of filter paper, and compressed using a meat press (Tenovo Meat-1; Tenovo International Co., Limited Beijing, China). The samples were reweighed (W4), water loss (%) = ([W3–W4]/W3) ×100%. Cooking loss, 50 g of the sample (W5) was weighed after the removal of tendons, fascia, and fat. The sample was boiled in water for 30 min, cooled for 30 min, and reweighed (W6), cooking loss (%) = ([W5–W6]/W5)×100%. Water holding capacity (WHC), samples 2.5 cm in diameter and 1 cm thick were cut and weighed (W7). The samples were pressed under a 35 kg weight for 5 min and reweighed (W8), WHC (%) = ([W7–W8]/W7)×100. Longitudinal sections of 1×1×3 cm parallel to the muscle fibers were cut and the shear force (in Newtons) was measured with a digital flexometer (Harbin Xeriki Co., Ltd., Harbin, China). Each sample was tested three times.

### Amino acid analysis

The preprocessing of BSF AA analysis followed the method outlined by Tian et al [14]. The UHPLC conditions for analyzing individual AA included using an ACQUITY UPLC BEH C18 column (2.1×100 mm×1.7 μm; Waters, Milford, MA, USA) at 40°C with a 5 μL injection volume. Mobile phases A and B consisted of 10% methanol (0.1% formic acid) and 50% methanol (0.1% formic acid), respectively. The elution gradient ranged from 10% to 30% B over 0 to 6.5 min, increased to 100% B from 6.5 to 7 min, held at 100% B from 7 to 8 min, decreased to 10% B from 8 to 8.5 min, and finally maintained at 10% B from 8.5 to 12.5 min. The flow rate changed from 0.3 mL/min to 0.4 mL/min during the run. Mass spectral analysis was conducted using an electrospray ion source in positive ion mode with a source temperature of 500°C, source voltage of 5,500 V, and gas pressures of 6 psi for impact gas, 30 psi for curtain gas, and 50 psi for atomization and auxiliary gases. Multi-reaction monitoring scan mode was used for data acquisition.

### Fatty acid profiles analysis

Individual LTL muscles were dried in a vacuum freeze dryer, and following the method by Tian et al [14], FA from both feed and muscle were extracted using a chloroform-methanol solution. The procedure was as follows: Approximately 50 mg of the sample was mixed with 3 mL of a chloroform-methanol solution (2:1) and agitated in a tissue lyser at 60 Hz for 15 min. The resulting extract was combined with 0.6 mL of physiological saline, followed by centrifugation at 4,000×g for 10 min to obtain a lipid extract. 1 mL of the lipid extract was mixed with 0.2 mL of a 5.00 mg/mL glycerol undecanoic acid triglyceride (C36H68O6, CAS: 13552-80-2) internal standard and esterified with 0.2 mL of methanol. This mixture was further esterified using 8 mL of a 2% sodium hydroxide-methanol solution. After adding 1 mL of n-heptane and centrifuging at 10,000×g for 5 min, the supernatant was collected, and 100 mg of powdered anhydrous sodium sulfate was added for drying. The dried extract was filtered through a 13 mm 0.45 μm nylon syringe filter and analyzed for individual fatty acids using gas chromatography-mass spectrometry (GC-MS) by Thermo Fisher Scientific. The instrumental conditions included a polydicyanopropylsiloxane strong polar stationary phase capillary column with a length of 100 m, inner diameter of 0.25 mm, and film thickness of 0.2 μm. The GC-MS parameters were set as follows: injector temperature at 270°C, detector temperature at 280°C, and a programmed temperature starting at 100°C for 13 min, followed by a heating rate of 10°C/min to reach 180°C and maintained for 6 min. Subsequently, the temperature was increased at a rate of 1°C/min to 200°C and held for 20 min, followed by a final increase to 230°C at a rate of 4°C/min and maintained for 10.5 min. The carrier gas used was nitrogen with a shunt ratio of 100:1, and the sample volume injected was 1.0 μL. The assay conditions aimed to achieve a theoretical plate number (n) of at least 2,000/m and a separation degree (R) of at least 1.25.

### Antioxidant analysis

The preparation of muscle homogenates followed the method by Wang et al [15], with slight modifications. In a glass homogenizer, mix 100 mg of muscle tissue with 900 mg of phosphate buffer solution. The mixture was then subjected to ultrasonication using a Bransonic ultrasonic cleaner (Branson Ultrasonics Corp., Danbury, CT, USA). Following ultrasonication, the muscle homogenate was collected by centrifugation at 4,000×g for 15 min and stored at –80°C until further analysis.

All the assay kits used in the analysis were obtained from Nanjing Jianjian Bioengineering Institute, with the following kit numbers: superoxide dismutase (SOD, #A001-2-2), catalase (CAT, #A007-1-1), glutathione peroxidase superoxide (GSH-Px, #A005-1-2), and total antioxidant capacity (T-AOC, #A015-1-2).

### Statistical analysis

Statistical analysis of data was performed in IBM SPSS Statistics 27 software (IBM, Armonk, NY, USA), with one-way analysis of variance among groups (followed by Fisher’s least significant difference post hoc comparison) for multiple comparisons. Statistical significance is defined when p-values are less than 0.05.

## RESULTS

### Slaughter performance and meat quality

Slaughtering performance and meat quality are shown in Table 5. The moisture, DM, CP, and ash content of the three muscle groups showed no significant differences. However, the EE content significantly increased (p<0.05) in the GRP_C_ and GRP_T_ groups. Dressing percentages significantly decreased (p<0.05) in the GRP_U_ and GRP_T_ groups. Comparatively, the GRP_U_ group led to lower (p<0.05) carcass weights compared to the GRP_C_ group. Additionally, drip loss and pH_45 min_ levels were significantly higher (p<0.05) in the GRP_U_ group compared to the GRP_C_ and GRP_T_ groups, with no significant differences observed between the GRP_C_ and GRP_T_ groups. No significant differences were observed among the three feeding groups in terms of shear force, WHC, cooking loss, and pH_24 h_. Furthermore, the L* value significantly increased (p<0.05) in the GRP_T_ group, while the a* and b* values significantly decreased (p<0.05).

### Amino acid content of *longissimus thoracis* and *lumborum*

Amino acids content of LTL is shown in Table 6. There was only the methionine level in the GRP_U_ group was significantly higher (p<0.05) than that in the GRP_C_ group. However, differences in other essential AA were non-significant in our study. There were no significant differences in the total amino acid (TAA), EAA, and umami amino acids (UAA) among the three feeding groups.

### Fatty acid content of *longissimus thoracis* and *lumborum*

Fatty acid content of LTL is shown in Table 7. The levels of C10:0, C18:1n9t, C18:2n6c, C20:0, and C18:3n3 in muscle were significantly reduced (p<0.05) in the GRP_U_ and GRP_T_ groups, while the levels of C14:1 and C16:1 were increased (p<0.05). Moreover, the levels of C12:0, C14:0, and C16:0 in the GRP_U_ group were significantly higher (p<0.05) than those in the GRP_C_ and GRP_T_ groups. The GRP_T_ group exhibited the highest (p<0.05) levels of C18:1n9c, while the GRP_U_ group showed the lowest levels (p<0.05). Additionally, the levels of C18:2n6t, C18:3n6, and C20:2 significantly increased (p<0.05) in the GRP_T_ group. The SFA levels were highest (p<0.05) in the GRP_U_ group, followed (p<0.05) by the GRP_C_ group, and lowest (p<0.05) in the GRP_T_ group. Furthermore, the GRP_U_ group significantly decreased (p<0.05) the PUFA/SFA ratio, but the n-6/n-3 ratio significantly increased (p<0.05) compared to the GRP_C_ group.

### Mineral content and antioxidant capacity of longissimus *longissimus thoracis* and *lumborum*

The mineral content and antioxidant capacity of longissimus LTL are shown in Table 8. There was only the Cu level in the GRP_U_ group was significantly lower (p<0.05) than that in the GRP_C_ group. There were no significant differences in the levels of other minerals and SOD in the muscles among the three feeding groups. The levels of CAT, GSH-Px, and T-AOC were significantly lower (p<0.05) in the GRP_U_ and GRP_T_ groups.

## DISCUSSION

Crude fat, CP, and moisture are the main chemical components of muscle. The fat content and fatty acid composition can influence the hardness of meat because different fatty acids have different melting points [16]. The water content of muscle directly influences the juiciness of the meat, while the crude ash content is related to the mineral and trace element content in the meat [17]. In this study, besides the significantly lower EE observed in the GRP_U_ group, there were no significant differences in moisture, DM, CP, and ash content. This suggests a weaker lipid deposition capacity in the muscles of the GRP_U_ group. Given the lack of research on the nutritional composition of ruminant muscle by BSF, and the rich protein and mineral content in chicken and goat meat, we have introduced a discussion on chicken in this section [18]. As Pieterse et al [19] reported, supplementing BSF had no significant impact on the approximate composition of broiler breast muscle. Aprianto et al [20] also obtained a similar result, suggesting that the BSF oil (1%, 2%, and 3%) feed supplement and approximate broiler muscle compositions were unrelated, however, with an increase in supplementation levels, there was a reduction in the deposition of EE in the muscle. Similarly, BSF reduced abdominal fat in laying hens [21] and broilers [22]. Many studies attributed this result to the high content of C12:0 in BSF because it was found that feeding palm oil, sunflower seed oil, flaxseed, or flaxseed oil resulted in higher intramuscular fat compared to animals fed coconut oil (also rich in C12:0) or BSF [23]. This interpretation was evidently in contrast with our study. Since the muscle EE content of GRP_T_ did not decrease but the levels of C12:0 in the diets of both GRP_U_ and GRP_T_ groups were similar. This could be attributed to the processing method of the GRP_T_ group, wherein starch, protein, and fatty acids undergo structural rearrangement at high temperatures, forming a protective barrier that reduced the oxidation of C12:0. According to reports, C12:0 was oxidized to CO_2_ faster than 18:3n-3 and other long-chain fatty acids, making it less available for storage in tissues or elongation to 14:0 and 16:0 [24].

Factors influencing meat quality include dressing percentage, color, pH, tenderness, WHC, and muscle fibers. Among them, the dressing percentage is an important index to evaluate carcass quality [25]. Meat color is a direct factor in assessing meat quality and customer purchasing intention. Muscle pH value is an important indicator reflecting the post-slaughter muscle glycolysis rate in animals. It is related to the WHC and color of goat meat [26]. In this study, the dressing percentage and carcass weight of BSF-fed goats were lower than those of the GRP_C_ group, indicating that BSF feeding might have a negative influence on carcass quality. A similar study result was also found by Murawska et al [27], BSF supplementation led to a decrease in the carcass yield of broiler chickens. This may be the increase of parts not intended for consumption, such as offal and limbs. Although shear force, WHC, and cooking loss showed no difference, the untreated BSF exhibited higher drip loss and pH_45 min_. Interestingly, the GRP_T_ group exhibited higher L* values, while a* and b* values were lower. This was inconsistent with the findings of research by Herrera et al [28], in which they concluded that BSF did not affect WHC, pH, and meat color in guinea pig muscles. Pieterse et al [19] also concluded that BSF did not impact the meat color and pH of chicken muscles. However, there was also a study that indicated that supplementing BSF increased the b* values and pH of chicken muscles [29]. Schiavone et al [30] observed a 10% increase in the a* value of chicken muscles with BSF supplementation, which is consistent with our GRP_U_ group results while in contrast with the GRP_T_ group. We assume that this may be related to the higher deposition of UFA in the LTL muscles of the GRP_T_ group. In summary, despite the lower EE content in the LTL of the GRP_U_ group, overall, the GRP_T_ group had a negative impact on meat quality.

The types and content of AA determine the nutritional value of proteins. In goat meat, the types and content of AA are related to factors such as animal species and feed composition. The results of this study indicated that there were no differences in TAA, EAA, and UAA among the three feeding groups. This is consistent with the findings of the studies conducted by Herrera et al [28]. The only exception was that the GRP_U_ exhibited higher levels of methionine compared to the GRP_C_ group. This may be related to the overall better meat quality in the GRP_U_ group, as methionine was positively correlated with muscle WHC, color stability, and the prevention of lipid and protein oxidation [31].

Monounsaturated fatty acids (MUFA) play a crucial role in reducing the incidence of coronary heart disease and preventing atherosclerosis, while PUFA is involved in the development of the brain and retina. Among PUFA, arachidonic acid (C20:4n6c), linseed oil, and alpha-linolenic acid (C18:3n3) are considered essential FA for the human body, which can only be obtained from food [32,33]. In this study, although the C18:3n3 content in the GRP_U_ and GRP_T_ groups was lower than that in the GRP_C_ group, the C18:1n9c in the GRP_T_ group was significantly higher than that in the GRP_C_ group, while the GRP_U_ group was significantly lower than the GRP_C_ group. Interestingly, a significant increase of UFA and a significant decrease of SFA in the GRP_T_ were observed, whereas the GRP_U_ group exhibited the opposite pattern. Moreover, in terms of the PUFA/SFA and n-6/n-3 ratios, the GRP_U_ group also demonstrated significantly lower values. Among individual SFA (C12:0 to C17:0), the content was consistently the lowest in the GRP_T_ group. However, no significant differences were observed in the content of these FA in the basic diets of the GRP_U_ and GRP_T_ groups. This indicated that GRP_T_ could improve the compositional pattern of FA in LTL muscles, promoting the deposition of FA in a more favorable direction. Our results regarding the positive influence of BSF on SFA was also confirmed by other reports [34,35]. In the GRP_T_ group, it may be that heat treatment forms starch-lipid complexes, which could protect the degradation of UFA in the rumen and allow more UFA to be absorbed in the small intestine [34], subsequently increasing the content of UFA in the muscles.

Generally, several minerals in meat such as Fe, Zn, Na, Ca, Mg, and Se are considered important for human intake. In this study, we observed that the Cu content in the LTL of the GRP_U_ group was significantly lower than that of the control group, while other minerals showed no significant differences. This indicated that most minerals in BSF were not absorbed and deposited in muscle tissue, possibly due to the lower bioavailability of minerals in insects [36]. Specific proteins (such as hemoglobin and casein) and mineral-binding peptides (casein phosphopeptides) support the absorption and bioavailability of minerals from traditional foods (such as meat and dairy). However, these pathways may not be applicable to the consumption of insects [37]. Furthermore, Cu transport occurs in two stages: first from the intestines to the liver and kidneys, and from the liver and kidneys to other tissues. This process requires the transport of albumin, transcuprein, and ceruloplasmin [38]. This study found that supplementation with BSF led to a decrease in antioxidant levels in muscle, which may indicate impaired liver and kidney function. Consequently, this resulted in a reduced deposition of Cu in the muscle of the GRP_U_ group.

The oxidation of meat can decrease its hydrolytic sensitivity, weaken protein degradation, and reduce the moisture reserves in myofibers, thereby increasing water loss and brightness in meat. Enhancing the antioxidant defense system can protect muscles from damage caused by free radicals, contributing to more effective binding and retention of moisture in the muscles [39]. However, this study found that the antioxidant levels in the GRP_U_ and GRP_T_ groups decreased. This contrasts with the results obtained by Dalle Zotte et al [24]. The decrease in muscle antioxidant levels in the GRP_T_ group could be attributed to the decline in meat quality and the increase in UFA contents. Furthermore, there was a downward trend in the levels of antioxidant-related minerals (Zn, Se, etc.) in the muscles of both GRP_U_ and GRP_T_, which may also contribute to the decrease in muscle antioxidant levels. In conclusion, supplementing BSF could be detrimental to the antioxidant performance of muscles.

## CONCLUSION

The current study demonstrated that there were no significant differences in muscle moisture, DM, CP, and ash among the three groups, but the EE of the GRP_U_ group was significantly reduced. Both the GRP_U_ and GRP_T_ groups exhibited a decrease in dressing percentage, carcass weight, and muscle antioxidant capacity. Additionally, the meat quality parameters of the GRP_T_ group were the poorest. Overall, there were no significant differences in the muscle AA and mineral content among the three groups. GRP_T_ improved the favorable FA composition in the muscle for human health, such as C18:2n6t, C18:3n6, and UFA. In summary, under the experimental conditions, both untreated and heat-treated BSF had some negative impacts on the quality of goat meat. However, heat-treated BSF demonstrated a positive effect on improving the FA composition of the muscle. Consequently, further development of various BSF treatment methods can be explored for their reasonable application in goat feed.

## Figures and Tables

**Table 1:** The proximate composition of black soldier fly (DM basis, %)

Items	Contents
DM	97.28
OM	87.74
CP	35.85
EE	37.65
Ca (g/kg)	45.90
Cu (mg/kg)	13.80
Fe (mg/kg)	886.00
K (g/kg)	14.30
Mg (g/kg)	3.51
Mn (mg/kg)	129.00
Na (g/kg)	3.08
P (g/kg)	8.48
Se (mg/kg)	0.50
Zn (mg/kg)	79.20

DM, dry matter; OM, organic matter; CP, crude protein; EE, ether extract.

**Table 2:** Concentrate supplement formula and chemical composition (DM basis, %)

Items	GRP_C_^1)^	GRP_U_^1)^	GRP_T_^1)^
Corn	64.0	64.0	64.0
Soybean meal	10.0	10.0	10.0
FFS	10.0	-	-
BSF	-	10.0	-
HTBSF	-	-	10.0
Wheat bran	10.0	10.0	10.0
Ca (H_2_PO_4_)_2_	1.0	1.0	1.0
NaCl	1.0	1.0	1.0
Premix^2)^	4.0	4.0	4.0
Total	100.0	100.0	100.0
Chemical composition (%)			
DM	94.79	95.19	95.19
OM	92.60	91.08	90.46
CP	16.10	16.19	16.09
EE	5.50	5.50	5.50
Ca	0.92	0.95	0.95
P	0.36	0.37	0.37
NDF	18.27	19.57	21.35
ADF	6.63	7.25	7.41
GE (KJ/g)	16.96	16.53	16.63

DM, dry matter; FFS, full-fat soybean; BSF, black soldier fly; HTBSF, heat-treated black soldier fly; OM, organic matter; CP, crude protein; EE, ether extract; NDF, neutral detergent fiber; ADF, acid detergent fiber; GE, gross energy.

1) GRP_C_, full-fat soybeans group; GRP_T_, heat-treated black soldier fly group; GRP_U_, black soldier fly group.

2) The vitamin-mineral premix was purchased from the Feed Division of Beijing Sanyuan Seed Industry Technology Co., Ltd. (Beijing, China), containing the following per kg: 100,000 IU of VA, 400 IU of VE, 11% of Ca, 1,500 mg/kg of Mg, 50 mg/kg of Cu, 1,500 mg/kg of Zn, 1,100 mg/kg of Mn, 20 mg/kg of P, 5 mg/kg of Se, 8 mg/kg of Co.

**Table 3:** Amino acids composition of concentrate

Items (mg/100 g)	GRP_C_^1)^	GRP_U_^1)^	GRP_T_^1)^
Indispensable amino acids			
Arginine	0.44±0.01	0.64±0.01	0.91±0.01
Histidine	-	-	-
Isoleucine	0.10±0.00	0.20±0.00	0.31±0.00
Leucine	0.89±0.00	1.10±0.01	1.21±0.00
Lysine	-	0.11±0.01	0.47±0.01
Methionine	-	-	-
Phenylalanine	0.13±0.04	0.45±0.01	0.63±0.00
Threonine	0.21±0.00	0.32±0.01	0.47±0.00
Valine	0.13±0.00	0.25±0.00	0.39±0.00
Dispensable amino acids			
Alanine	0.65±0.01	0.81±0.01	0.87±0.00
Aspartic acid	0.74±0.00	1.08±0.01	1.54±0.00
Glycine	0.39±0.00	0.56±0.00	0.70±0.00
Glutamic acid	2.05±0.01	2.62±0.01	3.00±0.01
Proline	0.72±0.01	0.79±0.04	0.91±0.00
Serine	0.40±0.00	0.57±0.00	0.75±0.00
Tyrosine	-	0.07±0.00	0.25±0.00

Values represent the means of twelve replicates (n = 3), mean±standard error.

1) GRP_C_, full-fat soybeans group; GRP_T_, heat-treated black soldier fly group; GRP_U_, black soldier fly group.

**Table 4:** Fatty acids composition of concentrate

Items (mg/100 g)	GRP_C_^1)^	GRP_U_^1)^	GRP_T_^1)^
C6:0	-	0.23±0.00	0.22±0.00
C8:0	-	-	0.08±0.00
C10:0	-	0.74±0.01	0.68±0.00
C12:0	0.08±0.00	16.32±0.01	15.51±0.01
C14:0	-	2.57±0.01	2.45±0.01
C14:1	-	0.05±0.00	-
C15:0	13.93±0.01	0.09±0.00	0.09±0.00
C16:0	0.14±0.00	16.82±0.01	17.41±0.01
C16:1	0.09±0.00	1.17±0.00	1.03±0.00
C17:0	2.93±0.01	0.23±0.00	0.23±0.00
C18:0	0.18±0.00	2.80±0.01	2.99±0.01
C18:1n9t	30.71±0.02	0.18±0.00	0.19±0.00
C18:1n9c	47.21±0.02	27.31±0.01	29.10±0.01
C18:2n6c	0.60±0.00	28.84±0.02	27.46±0.01
C20:0	0.29±0.00	0.34±0.00	0.40±0.00
C18:3n6	-	0.18±0.00	0.20±0.00
C20:1n9	2.87±0.00	-	0.31±0.00
C18:3n3	0.21±0.00	1.50±0.00	1.07±0.00
C21:0	0.31±0.00	-	-
C20:2	-	0.11±0.00	0.13±0.00
C20:4n6	0.22±0.00	0.07±0.00	-
C22:2	0.12±0.00	0.11±0.00	0.13±0.00
C24:0	0.10±0.00	0.18±0.00	0.17±0.00
C20:5n3	17.96±0.01	0.17±0.00	0.15±0.00
SFA	82.04±0.03	40.32±0.02	40.22±0.02
UFA	2.84±0.01	59.68±0.02	59.78±0.02
PUFA/SFA	2.97±0.01	0.72±0.00	0.72±0.00
n-3 PUFA	47.51±0.03	1.66±0.00	1.22±0.00
n-6 PUFA	30.89±0.02	29.09±0.01	27.66±0.01
n-9 PUFA	16.00±0.01	27.49±0.02	29.60±0.02
n-6/n-3	-	17.48±0.01	22.65±0.01

Values represent the means of twelve replicates (n = 3), mean±standard error.

SFA, saturated fatty acid; UFA, unsaturated fatty acid. PUFA, polyunsaturated fatty acid.

1) GRP_C_, full-fat soybeans group; GRP_T_, heat-treated black soldier fly group; GRP_U_, black soldier fly group.

**Table 5:** Effects of heat treatment black soldier fly on slaughter performance and meat quality

Items	GRP_C_^1)^	GRP_U_^1)^	GRP_T_^1)^	SEM	p-value
Moisture (%)	72.90	72.30	72.84	0.19	0.40
DM (%)	27.10	27.70	27.16	0.19	0.40
CP (%)	19.52	20.17	19.48	0.03	0.30
EE (%)	3.56^a^	3.40^b^	3.55^a^	0.19	0.02
Ash (%)	4.02	4.14	4.13	0.03	0.25
Dressing percentage (%)	49.53^a^	45.3^b^	45.15^b^	0.53	<0.01
Carcass weight (kg)	12.73^a^	11.02^b^	12.49^ab^	0.31	0.04
Shear force (N)	16.17	17.92	14.10	0.69	0.08
WHC (%)	87.16	85.04	86.72	0.88	0.61
Cooking loss (%)	42.84	42.12	43.20	0.41	0.59
Drip loss (%)	2.07^b^	2.56^a^	2.04^b^	0.09	0.04
pH_45 min_	6.10^ab^	6.19^a^	5.99^b^	0.03	0.04
pH_24 h_	5.46	5.70	5.53	0.04	0.75
Lightness (L*)	19.8^b^	25.2^ab^	26.6^a^	1.25	0.03
Redness (a*)	16.8^a^	17.4^a^	13.6^b^	0.49	<0.01
Yellowness (b*)	17.2^a^	17.3^a^	12.9^b^	0.58	<0.01

SEM, standard error of the mean; DM, dry matter; CP, crude protein; EE, ether extract; WHC, water holding capacity; pH_45 min_, pH at 45 min post-slaughter; pH_24 h_, pH at 24 h post-slaughter.

1) GRP_C_, full-fat soybeans group; GRP_T_, heat-treated black soldier fly group; GRP_U_, black soldier fly group.

a,b Different letters within a row are significantly different (p<0.05).

**Table 6:** Effects of heat treatment black soldier fly on amino acids content of muscle (mg/100 g)

Items	GRP_C_^1)^	GRP_U_^1)^	GRP_T_^1)^	SEM	p-value
Essential amino acids arginine					
Arginine	4.61	4.73	4.60	0.05	0.55
Histidine	2.24	2.26	2.42	0.04	0.11
Isoleucine	2.23	2.27	2.25	0.03	0.85
Leucine	5.94	6.04	5.96	0.07	0.83
Lysine	6.74	6.72	6.72	0.06	0.98
Methionine	1.32^b^	1.58^a^	1.51^ab^	0.04	0.04
Phenylalanine	3.08	3.17	3.17	0.04	0.60
Threonine	3.40	3.50	3.42	0.05	0.69
Valine	2.61	2.65	2.62	0.03	0.88
Dispensable amino acids					
Alanine	4.58	4.64	4.58	0.04	0.81
Aspartic acid	7.37	7.49	7.44	0.08	0.86
Glycine	3.62	3.70	3.66	0.03	0.51
Glutamic acid	12.14	12.50	12.43	0.14	0.58
Proline	2.91	2.99	2.78	0.05	0.20
Serine	3.30	3.45	3.32	0.05	0.47
Tyrosine	2.58	2.55	2.46	0.03	0.23
TAA	68.67	70.24	69.34	0.73	0.70
EAA	32.17	32.92	32.68	0.36	0.72
UAA	33.37	34.05	33.74	0.32	0.71

SEM, standard error of the mean; TAA, total amino acid; EAA, essential amino acid; UAA, umami amino acid (phenylalanine, alanine, aspartic acid, glycine, glutamic acid, tyrosine).

1) GRP_C_, full-fat soybeans group; GRP_T_, heat-treated black soldier fly group; GRP_U_, black soldier fly group.

a,b Different letters within a row are significantly different (p<0.05).

**Table 7:** Effects of heat treatment black soldier fly on fatty acids content of muscle (mg/100 g)

Items	GRP_C_^1)^	GRP_U_^1)^	GRP_T_^1)^	SEM	p-value
C10:0	0.18^a^	0.13^b^	0.11^b^	0.01	<0.01
C12:0	0.09^b^	0.15^a^	0.11^ab^	0.01	0.03
C14:0	1.69^b^	2.27^a^	1.82^b^	0.08	<0.01
C14:1	0.07^b^	0.11^a^	0.09^a^	0.001	<0.01
C15:0	0.35	0.42	0.32	0.02	0.08
C16:0	21.06^b^	23.38^a^	20.73^b^	0.31	<0.01
C16:1	1.37^b^	1.86^a^	1.82^a^	0.07	<0.01
C17:0	0.79^a^	0.80^a^	0.05^b^	0.13	0.02
C17:1	0.66	0.76	0.52	0.06	0.27
C18:0	15.67	15.39	15.53	0.23	0.90
C18:1n9t	1.79^a^	1.32^b^	1.26^b^	0.08	<0.01
C18:1n9c	51.75^b^	49.57^c^	53.62^a^	0.48	<0.01
C18:2n6t	0.23^b^	0.26^ab^	0.28^a^	0.01	0.03
C18:2n6c	3.65^a^	3.01^b^	3.06^b^	0.11	0.03
C20:0	0.18^a^	0.12^b^	0.09^b^	0.01	<0.01
C18:3n6	0.05^b^	0.03^b^	0.09^a^	0.01	0.04
C18:3n3	0.20^a^	0.15^b^	0.15^b^	0.01	<0.01
C20:2	-	0.02^b^	0.05^a^	0.01	<0.01
C20:3n6	-	-	0.01	0.00	-
C20:4n6	0.30	0.27	0.28	0.02	0.83
SFA	39.99^b^	42.66^a^	38.76^c^	0.44	<0.01
UFA	60.01^b^	57.34^c^	61.24^a^	0.44	<0.01
PUFA/SFA	0.11^a^	0.09^b^	0.10^a^	0.003	<0.01
n-3 PUFA	0.15	0.15	0.15	0.01	0.98
n-6 PUFA	4.20	3.56	3.73	0.12	0.06
n-9 PUFA	53.54^a^	50.89^b^	54.88^a^	0.47	<0.01
n-6/n-3	29.81^a^	24.43^b^	24.79^ab^	0.95	<0.01

SEM, standard error of the mean; SFA, saturated fatty acid; UFA, unsaturated fatty acid; PUFA, polyunsaturated fatty acid.

1) GRP_C_, full-fat soybeans group; GRP_T_, heat-treated black soldier fly group; GRP_U_, black soldier fly group.

a–c Different letters within a row are significantly different (p<0.05).

**Table 8:** Effects of heat treatment black soldier fly on the mineral content and antioxidant capacity of *longissimus thoracis* and *lumborum*

Items	GRP_C_^1)^	GRP_U_^1)^	GRP_T_^1)^	SEM	p-value
Fe (mg/kg)	67.99	59.95	56.46	3.14	0.40
Mn (mg/kg)	0.58	0.42	0.44	0.03	0.11
Cu (mg/kg)	2.84^a^	2.30^b^	2.58^ab^	0.09	0.04
Zn (mg/kg)	109.18	102.87	102.89	2.18	0.50
K (g/kg)	19.43	18.80	19.27	0.20	0.51
Na (g/kg)	2.04	2.21	2.09	0.06	0.62
Ca (mg/kg)	258.30	196.75	239.72	13.89	0.23
Mg (g/kg)	1.17	1.13	1.18	0.01	0.34
P (g/kg)	9.68	9.30	9.54	0.10	0.38
Se (mg/kg)	0.46	0.43	0.45	0.01	0.47
SOD (U/mL)	88.13	94.45	88.69	1.29	0.08
CAT (U/mL)	13.87^a^	5.72^b^	6.71^b^	0.92	<0.01
GSH-Px (U/mL)	54.29^a^	47.13^b^	48.89^b^	0.84	<0.01
T-AOC (mmol/mL)	16.00^a^	13.64^b^	14.02^b^	0.31	<0.01

SEM, standard error of the mean; SOD, superoxide dismutase; CAT, catalase; GSH-Px, glutathione peroxidase superoxide; T-AOC, total antioxidant capacity.

1) GRP_C_, full-fat soybeans group; GRP_T_, heat-treated black soldier fly group; GRP_U_, black soldier fly group.

a,b Different letters within a row are significantly different (p<0.05).
